# Application of propionate-producing bacterial consortium in ruminal methanogenesis inhibited environment with bromoethanesulfonate as a methanogen direct inhibitor

**DOI:** 10.3389/fvets.2024.1422474

**Published:** 2024-10-09

**Authors:** Jongsik Jeong, Chaemin Yu, Ryukseok Kang, Myunghoo Kim, Tansol Park

**Affiliations:** ^1^Department of Animal Science and Technology, Chung-Ang University, Anseong, Republic of Korea; ^2^Department of Animal Science, College of Natural Resources and Life Science, Pusan National University, Miryang, Republic of Korea; ^3^Institute for Future Earth, JYS Institute for Basic Science, Pusan National University, Pusan, Republic of Korea

**Keywords:** ruminant, methane emission, methanogen direct inhibition, probiotics, alternative hydrogen sink, propionate production

## Abstract

Methane production in ruminants is primarily due to the conversion of metabolic hydrogen (H_2_), produced during anaerobic microbial fermentation, into methane by ruminal methanogens. While this process plays a crucial role in efficiently disposes of H_2_, it also contributes to environmental pollution and eliminating methane production in the rumen has proven to be challenging. This study investigates the use of probiotics, specifically propionate-producing bacteria, to redirect accumulated H_2_ in a methane-mitigated environment. For this objective, we supplemented experimental groups with *Lactiplantibacillus plantarum* and *Megasphaera elsdenii* for the reinforced acrylate pathway (RA) and *Selenomonas ruminantium* and *Acidipropionibacterium thoenii* for the reinforced succinate pathway (RS), as well as a consortium of all four strains (CB), with the total microbial concentration at 1.0 × 10^10^ cells/mL. To create a methane-mitigated environment, 2-bromoethanesulfonate (BES) was added to all experimental groups at a dose of 15 mg/0.5 g of feed. BES reduced methane production by 85% *in vitro*, and the addition of propionate-producing bacteria with BES further decreased methane emission by up to 94% compared with the control (CON) group. Although BES did not affect the alpha diversity of the ruminal bacteriome, it reduced total volatile fatty acid production and altered beta diversity of ruminal bacteriota, indicating microbial metabolic adaptations to H_2_ accumulation. Despite using different bacterial strains targeting divergent metabolic pathways (RA and RS), a decrease in the dominance of the [*Eubacterium*] *ruminantium* group suggesting that both approaches may have a similar modulatory effect. An increase in the relative abundance of *Succiniclasticum* in the CB group suggests that propionate metabolism is enhanced by the addition of a propionate-producing bacterial consortium. These findings recommend using a consortium of propionate-producing bacteria to manage H_2_ accumulation by altering the rumen bacteriome, thus mitigating the negative effects of methane reduction strategies.

## Introduction

The generation of methane gas (CH_4_) in ruminants is an excretory process that has evolved over a long time as a result of microbial fermentation in the rumen, making it impossible to completely inhibit CH_4_ production (1). Methanogenesis in the rumen causes a significant loss of gross energy (2–12%) and far greater global warming than does carbon dioxide (CO_2_), causing a negative impact on the environment (2). These factors underscore the critical need for strategies to reduce CH_4_ emissions from ruminant livestock. Several methods to mitigate CH_4_ emissions, such as defaunation (3), the use of probiotics (4, 5), feed additives, including plant extracts (6–8), bacteriocins (9), nitrates and sulfates (10), fumarates (11, 12), synthetic compounds like 3-nitrooxypropanol (13, 14), bromo-organic compounds from seaweed (15, 16), and ionophores like monensin (17), and adjustments in feed composition, including altering the forage-to-concentrate ratio (18) and supplementing dried distillers grains with solubles (19), have been employed. These strategies can be broadly classified into the following categories based on their mechanisms: indirect and direct approaches (20, 21). Indirect mechanisms aim to reduce the substrates available for methanogens by inhibiting fibrolytic bacteria, protozoa, or fungi (22). While effective in reducing methane, these microorganisms are essential for the digestion of fibrous feed, and their inhibition can adversely affect animal nutrition and productivity. Conversely, direct strategies aim to suppress the population of methanogenic archaea directly, thus significantly lowering methane production. Although this method seems optimal, it presents challenges in maintaining animal productivity. Suppressing methanogens disrupts normal microbial fermentation (23, 24), leading to the accumulation of hydrogen (H_2_), which in turn hinders the re-oxidation processes of NADH, NADPH, and FADH_2_. This reduction may potentially decrease the production of volatile fatty acids (VFAs) (25, 26), resulting in microbial adaptation that could eventually counteract methane reduction efforts. Therefore, managing H_2_ concentration when using direct methanogen inhibitors is a crucial factor that must be carefully considered and addressed.

Hydrogenotrophic bacteria that induce H_2_ uptake through alternative H_2_ sinks, such as dissimilatory sulfate reduction, nitrate reduction, propionate production, and reductive acetogenesis, compete with ruminal methanogens (27). However, the reduced efficiency of H_2_-capture by propionate producers and reductive acetogens, combined with the limited availability of electron acceptors for nitrate and sulfate reducers, makes it difficult for these hydrogenotrophic bacteria to dominate in the rumen (10, 24, 27). Thus, in an environment where ruminal methanogens are reduced by specific inhibitors, hydrogenotrophic bacteria with lower H_2_ affinity may benefit from the absence of their dominant competitors. Moreover, the increased H₂ concentration resulting from methanogen-specific inhibitors can enhance the availability of H₂ and provide an opportunity to boost the dominance of hydrogenotrophic bacteria (24).

VFAs, including acetate, propionate, and butyrate, are key end-products of microbial fermentation in the rumen, serving as significant energy sources for ruminants and substrates for methanogenesis (28). Unlike the acetate and butyrate production pathways, the propionate production pathway consumes a greater amount of hydrogen than it generates. As a result, it functions as a net hydrogen sink, which may diminish the availability of hydrogen for methanogenesis through competitive interactions (1). Efforts to enhance propionate production, aiming to limit methanogenesis by leveraging this competitive mechanism, have been explored (27, 29, 30). Despite these efforts, the affinity of propionate producers for H₂ is lower than that of methanogens, thus maintaining the dominance of methanogens and limited methane reduction, which was typically restricted to approximately 10–15% in normal rumen conditions (1).

A comparative study of gene expression patterns associated with H₂ metabolism between high- and low-methane-emitting animals (31) revealed similar expression levels of enzymes involved in H₂ production. However, enzymes mediating various alternative H_2_ sinks that induce H₂ uptake were activated in low-methane-emitting animals. Additional research has shown increased gene and transcript abundance related to lactate and propionate production as well as butyrate conversion from acetate, which all contribute to H₂ uptake in low-methane-emitting animals (32, 33). Based on these findings, increasing the dominance of microbes that contribute to alternative H₂ sinks, such as propionate production in the rumen, to induce H₂ uptake appears to be a feasible approach for methane mitigation.

Considering these factors comprehensively, we hypothesized that using a bacterial consortium focused on the propionate production pathway (24, 34), combined with the application of a direct methanogen inhibitor (2-bromoethanesulfonate, BES) (35–37) will not only aid in addressing the issue of accumulated H₂ but also help in further reducing methane production. Furthermore, we suggest that utilizing a consortium of bacteria involved in the propionate pathway (34) rather than individual strains could more effectively modulate the rumen microbiome. This strategy promotes pathway-based enhancement and represents a synergistic approach that could be practically applied on farms to achieve a more substantial reduction in methane emissions.

## Materials and methods

### Selection and preparation of microbes

The selection of bacterial strains for the *in vitro* fermentation experiment was based on their involvement in the metabolic pathways responsible for propionate production in the rumen (acrylate and succinate pathways) (Table 1). *Lactiplantibacillus plantarum* (KCTC 3103), *Megasphaera elsdenii* (KCTC 5187), and *Acidipropionibacterium thoenii* (KCTC 5343) were obtained from the Korean Collection for Type Cultures (KCTC, Jeongeup, Republic of Korea). *Selenomonas ruminantium* (DSM 2150) was obtained from the German Collection of Microorganisms and Cell Cultures GmbH (DSMZ; Braunschweig, Germany). *L. plantarum* is a transient, facultative anaerobic bacterium, which is generally introduced through the food in the rumen (38). *L. plantarum* has the potential for lactate production to facilitate the acrylate pathway for propionate production (34) and reduction in methanogenesis by inoculation of *L. plantarum* BX62 strain (39). *M. elsdenii*, *A. thoenii*, and *S. ruminantium* are obligate anaerobic bacteria residing in the rumen. *M. elsdenii* is involved in the acrylate pathway and has been reported to produce not only butyrate by fermenting glucose but also propionate through lactate fermentation (1, 40, 41). Among the various propionic acid bacterial strains, *A. thoenii* has a potential for methane mitigation (30). *S. ruminantium* is reported as a propionate producer and succinate utilizer (27). Additionally, *A. thoenii* and *S. ruminantium* can use lactate as a substrate for propionate production (27). These bacterial strains were cultured in reinforced clostridial medium (RCM) supplemented with 10% clarified rumen fluid under strict anaerobic conditions. The cultures were preserved at −80°C in 20% glycerol. For the *in vitro* fermentation experiment, 1% volume aliquots (100 μL) of each strain’s frozen stock were inoculated into RCM with 10% clarified rumen fluid and incubated at 37°C under strict anaerobic conditions. Incubation times were 48 h for all strains except for *L. plantarum*, which was incubated for 24 h. Microbial growth was quantified and standardized to an optical density of 1.0 at 600 nm (OD_600_). Cultures achieving this growth level were temporarily stored at 4°C for up to 12 h before cell harvesting. Considering the inverse relationship between cell concentration and cell volume at an OD of 1.0 (42), the cell volume was evaluated by following instructions in Bergey’s manual (43, 44) and a previous study (45). Subsequently, the desired cell quantities for each strain were established using the calculated cell volume (46) to precisely ascertain the required cell counts for every strain. While the reinforced acrylate pathway (RA) and reinforced succinate pathway (RS) group each requires a cell count of 5.0 × 10^9^ cells/mL per species, the consortium group (CB) group requires one of 2.5 × 10^9^ cells/mL. For cell harvesting, cultures were transferred to 2-mL screw cap tubes equipped with O-rings in an anaerobic workstation (Whitley DG250; Don Whitley Scientific, England) and centrifuged at 10,000 *g* for 10 min at 4°C. After centrifugation, the supernatant was removed, and the cells were washed twice with an anaerobic salt solution (comprising K_2_HPO_4_ 1.0 g/L, KH_2_PO_4_ 1.0 g/L, NaHCO_3_ 10.0 g/L, NaCl 2.0 g/L, MgSO_4_·7H_2_O 0.5 g/L in anoxic solution adjusted to pH 6.5–6.7) to remove the residual medium components. The final cell suspension volume was adjusted to 1 mL per tube by combining each bacterial strain within their respective groups using the same anaerobic salt solution, in preparation for inoculation. Prepared bacterial inoculants were stored at 4°C until use, removed 30 min before inoculation to allow temperature equilibration, and then introduced into fermentation bottles as part of the inoculation procedure.

**Table 1:** Bacterial strains used in the *in vitro* fermentation experiment for enhancing the propionate production pathway.

Bacterial strain	Associated pathway	Group	
*Lactiplantibacillus plantarum*	Acrylate–lactate production	RA	CB
*Megasphaera elsdenii*	Acrylate–propionate production		
*Acidipropionibacterium thoenii*	Succinate–propionate production	RS	
*Selenomonas ruminantium*	Succinate–propionate production		

Standardization of total cell number: 1.0 × 10^10^ cells/mL for the reinforced acrylate (RA) pathway group, 5.0 × 10^9^ cells/mL of *L. plantarum* (KCTC 3103) and *M. elsdenii* (KCTC 5187); for the reinforced succinate (RS) pathway group, 5.0 × 10^9^ cells/mL of *S. ruminantium* (DSM 2150) and *A. thoenii* (KCTC 5343); for the consortium (CB) group, 2.5 × 10^9^ cells/mL of *L. plantarum*, *M. elsdenii*, *S. ruminantium*, and *A. thoenii*.

### Experimental design

The *in vitro* fermentation experiment was structured based on a completely randomized design, with each of the treatment groups replicated five times. The groups were organized as follows: (1) CON, which served as the control group; (2) BES, 15 mg of 2-bromoethanesulfonate per 0.5 g of basal diet was added (137502-25G, Sigma-Aldrich, St. Louis, MO, USA) (35); (3) RA, the reinforced acrylate pathway group, which included the same BES concentration along with *L. plantarum* and *M. elsdenii*; (4) RS, the reinforced succinate pathway group, which included the same BES concentration along with *S. ruminantium* and *A. thoenii*; and (5) CB, the consortium group for propionate production pathway, incorporating the same concentration of BES and all four microbial strains. BES was included in all the treatment groups except the CON group to assess its effectiveness in creating a methane-reducing environment. The overall microbial dose for the groups receiving microbial supplementations (RA, RS, and CB) was based on the estimated cell counts and then set at 1.0 × 10^10^ cells/mL to ensure a consistent level of microbial influence across these treatments.

### *In vitro* fermentation experiment

For the *in vitro* fermentation experiment, the stomach tubing method was used to collect rumen fluid from three Hanwoo cows located at the Nonghyup Co., Ltd. research farm. The time required to reach our laboratory from this farm is approximately 30 min. The procedures were authorized by the Institutional Animal Care and Use Committee (IACUC) at Chung-Ang University (202401030036) and conducted in compliance with the ethical standards outlined in the IACUC guidelines for animal welfare. Collection was performed before the morning feeding at 7:30 a.m. To ensure the preservation of sample integrity, the rumen fluid was transported to the laboratory within 30 min in an airtight, insulated container pre-flushed with 99.999% CO_2_ gas, which was passed through a pre-heated copper column to maintain a stable temperature and anaerobiosis. Upon arrival at the laboratory, the rumen fluid underwent filtration through two cheesecloth layers to eliminate particulate impurities. Subsequently, the filtered rumen fluid was combined with a buffer solution at twice its volume, as specified previously (47), and the mixed inoculum’s pH was adjusted to 6.7. The inoculum was continuously flushed with oxygen-free CO_2_ to maintain anaerobic conditions. The experimental substrate was the same as the feed used at the research farm [comprising pellet concentrate and oat hay (Table 2)]. It was ground through a 1.0-mm sieve and oven-dried at 55°C for 48 h before being placed in the fermentation bottles. Each 125-mL fermentation bottle was loaded with 0.5 g of this feed with a forage-to-concentrate ratio of 1:1 and 50 mL of the prepared inoculum. All procedures were performed under anaerobic conditions. After inoculating 1 mL of the pre-prepared microbial tube, the inoculated bottles were immediately sealed with butyl rubber stoppers and aluminum caps and placed in a shaking incubator. The incubator conditions were set to 39°C with a shaking speed of 60 rpm, and the samples were incubated for 48 h.

**Table 2:** Composition of diet used in the *in vitro* fermentation experiment.

Item (% of DM)	Oat hay	Pellet concentrate
Dry matter	91.56	89.19
Crude protein	3.55	16.20
Crude fat	2.15	4.08
Crude fiber	27.40	8.66
Crude ash	5.02	6.95
Calcium	0.09	1.22
Phosphorus	0.09	0.65
ADF	30.87	13.23
NDF	54.17	31.10

DM, dry matter; ADF, acid detergent fiber; NDF, neutral detergent fiber.

### Sample collection and fermentation measurements

After incubation, total gas and CH_4_ production, pH, ammonia nitrogen (NH_3_-N), VFA concentration, dry matter digestibility (DMD), and neutral detergent fiber digestibility (NDFD) were analyzed. The headspace gas pressure of the fermentation bottle was recorded using a pressure transducer (L20000DCV3, Laurel Electronics, Inc., Costa Mesa, CA, USA) and simultaneously collected in a gas bag using a rubber stopper, needle, and 3-way cock setup (48). Gas pressure was recorded and collected at intervals of 3, 6, 12, 24, 36, and 48 h during incubation (49), with continuous monitoring to ensure that the pressure did not exceed 480 mbar and prevent microbial growth inhibition (50). Gas volume was estimated by converting mbar readings into mL by injecting air into a similarly sized fermentation bottle filled with distilled water (DW) and an equal volume of inoculum for calibration under standard atmospheric pressure (48). CH_4_ concentration from the collected gas (2 mL) was determined using gas chromatography (YL6500 GC system, Youngin Chromass, Anyang, Republic of Korea) equipped with a thermal conductivity detector and a packed GC column (G3591-80055, Agilent Technologies Inc., Santa Clara, CA, USA), employing a gas-tight syringe (1010 TLL, Hamilton company, Reno, NV, USA) for injection. CH_4_ measurements were taken in duplicates within 2 days of collection. After 48 h of incubation using a cut tip, 1.8 mL of inoculum was pipetted into a 2-mL microcentrifuge tube. Subsequently, the residual inoculum was transferred to a 50-mL conical tube for pH analysis using a pH meter (MW150, Milwaukee Instruments, Inc., Rocky Mount, NC, USA). The inoculum was then placed in a pre-weighed R510 nylon bag (Ankom Technology, Macedon, NY, USA), carefully transferring the remaining particles with DW. The bag was gently squeezed 2–3 times before drying in an oven at 65°C for 72 h to measure DMD, ensuring all sampling procedures were carefully conducted using ice to prevent additional microbial fermentation and to avoid unnecessary DM loss. For VFA and NH_3_-N analyses, samples were prepared by centrifuging 1.8 mL of the inoculum at 16,000 *g* for 15 min at 4°C. For VFA analysis, 1.0 mL was mixed with 0.2 mL of 25% (w/v) metaphosphoric acid; for NH_3_-N analysis, 0.5 mL was mixed with 0.1 mL of 0.2 M sulfuric acid (H_2_SO_4_). VFA samples were stored at −80°C and NH_3_-N samples, at 4°C. The remaining pellet was stored at −80°C to use later for extraction during quantitative real-time polymerase chain reaction (qPCR) and microbiome analyses. VFA concentration was quantified using gas chromatography (7890B, Agilent Technologies Inc., Santa Clara, CA, USA) with an autosampler (7693A, Agilent Technologies Inc., Santa Clara, CA, USA), flame ionization detector, and a capillary column (Nukol Fused silica capillary column, Supelco, Bellefonte, PA, USA). The carrier gas was nitrogen (N_2_), with a makeup flow set at 30 mL/min and a column flow set at 1 mL/min. The detector and inlet temperatures were set to 220°C. The initial oven temperature was set to 90°C, which was then increased to 200°C at a rate of 15°C/min, followed by a hold time of 2 min. Subsequently, the temperature was raised to 220°C at a rate of 20°C/min, with a final hold time of 3 min for measurement. The split ratio was set at 10:1, and the injection volume was 0.8 μL. NH_3_-N concentration was determined using a micro plate spectrophotometer (INNO, LTEK, Seongnam, Republic of Korea) by following the colorimetric method of Chaney and Marbach (51) with modifications by Hamid et al. (49) using ammonium chloride as a standard. After the DMD analysis, the maximum amount of DM in the R510 nylon bag was transferred to a F57 filter bag (Ankom Technology, Macedon, NY, USA) for NDFD measurement using a fiber analyzer (A200, Ankom Technology, Macedon, NY, USA), in accordance with the Ankom NDF procedure and incorporating the use of heat-stable *α*-amylase.

### Metagenomic DNA extraction

Total metagenomic DNA was extracted from the microbial pellets using the repeated bead-beating plus column purification (RBB + C) method by following a previously described protocol (52). For the preparation of qPCR standards, genomic DNA from the pure cultures of the four bacterial strains was extracted using the AccuPrep^®^ Genomic DNA Extraction Kit (Bioneer, Daejeon, Republic of Korea). This process also incorporated mechanical lysis with bead-beating to ensure a thorough breakdown of cell walls for optimal DNA yield. Additional qPCR standards were prepared from aliquots of the extracted metagenomic DNA samples, ensuring a broad representation of the microbial DNA present in the samples for accurate quantification.

### High-fidelity long-read sequencing

For the analysis of the bacteriota, samples were amplified and sequenced at Macrogen Inc. (Seoul, Republic of Korea) using the HiFi long-read sequencing platform PacBio Sequel IIe system (Pacific Biosciences, CA, USA). The 16S rRNA gene bacterial universal primer pairs used were 27F (5′-AGRGTTYGATYMTGGCTCAG-3′) and 1492R (5′-GYTACCTTGTTACGACTT-3′). The resultant raw fastq files were then imported into QIIME2 environment^1^ (53) for comprehensive microbiome analysis. After demultiplexing, the sequences were quality-filtered, and the denoising process, which involves removing primer and adaptor sequences as well as eliminating chimeric sequences, was performed using the DADA2 plugin (54). Amplicon sequence variants (ASVs), with a mean length of 1,457 nucleotides, were taxonomically classified using the Silva 138 99% operational taxonomic units (OTUs) reference database (55) with the confidence threshold set at 0.8. The minimal sequence count across all samples was 19,752, and repeated rarefaction was conducted 1,000 times at this minimum sequence count to normalize data across the samples (56). The differences in rumen bacterial diversity within individual samples (alpha diversity) and comparative diversity between samples (beta diversity) were analyzed based on the repeatedly rarefied ASV table. Beta diversity was visualized using non-metric multidimensional scaling (NMDS) plots based on the Bray–Curtis dissimilarity distance matrices.

### Quantitative real-time PCR

Primer sets for the quantification of specific microbial populations were chosen based on previous studies (see Supplementary Table 1). Unique gene regions were targeted for *L. plantarum* and *A. thoenii*, while the primer sets for other microbes primarily focused on 16S rRNA gene sequences, except the 18S rRNA gene sequence used for protozoa. Initial conventional PCR amplifications were performed to generate qPCR standards using 1 μL genomic DNA per reaction in a PCR thermal cycler (TP 600, TaKaRa, Kusatsu, Gunma, Japan). The amplification conditions, including cycling parameters and temperature settings, were adapted from established protocols corresponding to each primer set. The specificity of each primer set was verified and confirmed using TestPrime^2^ (57) and confirmed by gel electrophoresis on a 1.5% agarose gel. After verification, the PCR products were purified using the AccuPrep PCR/Gel Purification Kit (Bioneer, Daejeon, Republic of Korea). The concentration of nucleic acids in the purified amplicons was measured using a NanoDrop One microvolume UV–Vis spectrophotometer (Thermo Fisher Scientific, Wilmington, NC, USA), facilitating the calculation of copy numbers per mL for standard curve preparation. These standards were subsequently stored at −20°C until needed. Microbial quantification was performed on the QuantStudio 1 system (Thermo Fisher Scientific, Wilmington, NC, USA). Each qPCR reaction comprised 1 μL template DNA (genomic or metagenomic) added to 15 μL reaction mixture. This mixture included 0.075 μL of each primer (forward and reverse, both at 100 μM concentration), 7.5 μL PowerUp SYBR Master Mix (2X), and 6.35 μL ultra-pure water. For total protozoa quantification, 2 μL template DNA was used. The qPCR amplification protocol was executed in accordance with the specific guidelines for each primer set, ensuring accurate and reliable microbial quantification.

### Statistical analyses

The Shapiro–Wilk test was conducted to examine whether microbial fermentation parameters followed a normal distribution. Levene’s test was utilized to assess the equality of variances among the groups. Such parameters were then analyzed using the Proc Glimmix Procedure in SAS, version 9.4 (SAS Institute, Cary, NC, USA), incorporating Tukey’s honestly significant difference (HSD) test to account for supplementation effects as a fixed effect. Conversely, for data sets, including total gas production, pH, methane yield (measured in mL/g of degraded dry matter [dDM]), and the acetate-to-propionate ratio (A:P ratio), which diverged from normal distribution, the non-parametric Kruskal–Wallis H test followed by Dunn’s post-hoc test, was applied. Significance of the supplementation effects was declared at *p* ≤ 0.05, while tendency was noted when 0.05 < *p* ≤ 0.10. Microbial diversity analysis encompassed alpha diversity metrics, such as Chao1, Simpson, Shannon, and Faith’s Phylogenetic Diversity, processed in QIIME2 (53). The diversity indices were analyzed using the Proc Glimmix Procedure (SAS, version 9.4, SAS Institute, Cary, NC, USA) with Tukey’s HSD test. Beta diversity was analyzed using permutation analysis of variance (PERMANOVA) tests, executed 9,999 times using the adonis2 function within the vegan package in R (58), with results corrected for multiple comparisons using the Benjamini–Hochberg method based on the Bray–Curtis dissimilarity distance matrix. To investigate the multivariate association in the ruminal bacteriome, microbial compositional data were analyzed using MaAsLin 2 (59), focusing on major taxa present in all groups at 100% occurrence. Significant differences were determined based on a false discovery rate-adjusted *p*-value (*Q*-value) of ≤0.05 by applying the Benjamini–Hochberg procedure for multiple test corrections. Pearson correlation analysis was conducted using PROC CORR in SAS, version 9.4 (SAS Institute, Cary, NC, USA) with the results visualized in a heatmap generated in R, facilitating the interpretation of correlations between microbial fermentation parameters and differentially abundant microbial relative abundances, while for the supplemented bacterial strains, copy numbers were used instead of relative abundances.

## Results

### Fermentation characteristics

**Figure 1:**
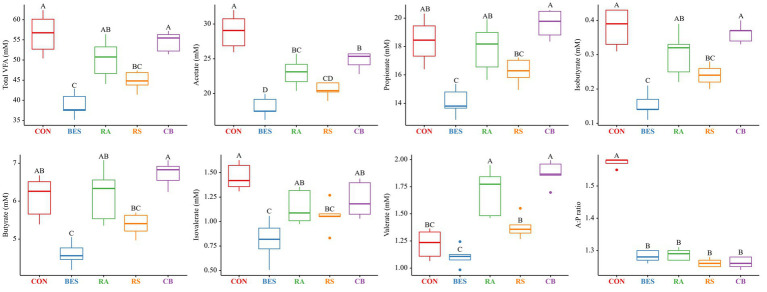
VFA characteristics after 48 h of the *in vitro* fermentation experiment. BES was added to the RA, RS, and CB groups at the same concentration as that (15 mg/0.5 g) in the BES group. The RA group comprised *Lactiplantibacillus plantarum* and *Megasphaera elsdenii*; RS group, *Selenomonas ruminantium* and *Acidipropionibacterium thoenii*; and CB group, all four bacterial strains. CON, control group; BES, 2-bromoethanesulfonate group; RA, reinforced acrylate pathway group; RS, reinforced succinate pathway group; CB, propionate-producing bacterial consortium group; VFA, volatile fatty acid; A:P ratio, acetate-to-propionate ratio. ^A–D^: significant differences (*p* ≤ 0.05) between treatment groups.

The results from the 48-h *in vitro* fermentation experiment, detailing the fermentation characteristics, are summarized in Table 3 and Figure 1. The incorporation of BES markedly decreased total gas production (*p* < 0.0001); however, it had no significant impact on DMD, NDFD, and pH. The NH_3_-N levels showed a tendency to decrease in the RA group. The supplementation of propionate-producing bacteria in conjunction with BES (in the RA, RS, and CB groups) led to a greater reduction in methane production (*p* < 0.0001) than that in the BES-only group. Although BES addition initially lowered total VFA production significantly (*p* < 0.0001), the incorporation of some propionate-producing bacteria (particularly in the RA and CB groups) was found to restore VFA levels that were comparable with those in the CON group. Specifically, the acetate concentration significantly increased in the RA and CB groups with microbial supplementation, although these levels did not fully match those of the CON group. However, the levels of other VFAs, such as propionate and butyrate, were restored to those observed in the CON group with RA and CB supplementation. The valerate concentration was significantly higher in the RA and CB groups than that in the CON group. Overall, the addition of propionate-producing bacteria along with BES not only increased the methane mitigation potential but also restored the VFA patterns disrupted by BES treatment.

**Table 3:** Fermentation parameters measured over 48 h in the *in vitro* fermentation experiment.

Parameter	Group					SEM	*p*-value
	CON	BES	BES (+)				
			RA	RS	CB		
DMD (%)	67.54	67.45	67.54	66.95	68.48	4.47	0.6725
NDFD (%)	54.34	54.36	55.20	54.90	52.30	5.94	0.4296
pH	6.50	6.50	6.50	6.51	6.50	3.10	0.4399
NH_3_-N (mg/dL)	22.73^a^	20.57^ab^	20.32^b^	22.00^ab^	21.18^ab^	4.11	0.0573
Total gas (mL)	115.97^A^	103.54^B^	102.16^B^	101.56^B^	102.21^B^	4.72	<0.0001
CH_4_ (mL)	4.58^A^	0.66^B^	0.27^C^	0.28^C^	0.33^C^	3.11	<0.0001
CH_4_ (mL/g dDM)	13.56^A^	1.97^B^	0.80^C^	0.85^C^	0.97^C^	3.23	<0.0001

BES was added to the RA, RS, and CB groups at the same concentration as that (15 mg/0.5 g) in the BES group.

The RA group comprised *Lactiplantibacillus plantarum* and *Megasphaera elsdenii*; RS group, *Selenomonas ruminantium* and *Acidipropionibacterium thoenii*; and CB group, all four bacterial strains.

CON, control group; BES, 2-bromoethanesulfonate group; RA, reinforced acrylate pathway group; RS, reinforced succinate pathway group; CB, propionate-producing bacterial consortium group; CH_4_ (mL/g dDM), quantity of methane production per degraded gram of dry matter; NH_3_-N, ammonia nitrogen; DMD, dry matter digestibility; NDFD, neutral detergent fiber digestibility; SEM, pooled standard error of the mean.

^A–C^: significant differences (*p* ≤ 0.05) between treatment groups.

^a–b^: tendency (0.05 < *p* ≤ 0.10) between treatment groups.

### Diversity analyses

**Figure 2:**
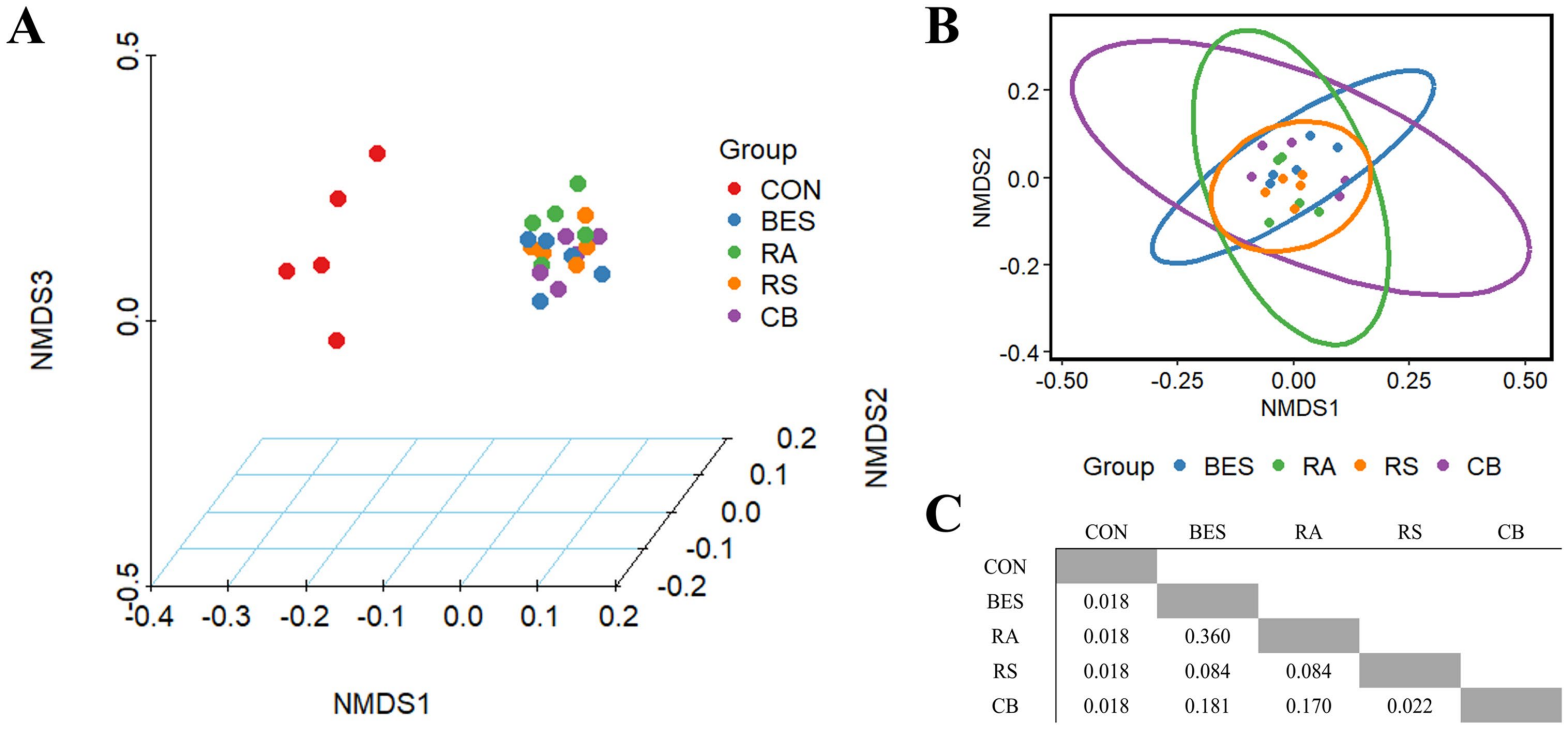
NMDS plot based on Bray-Curtis distance matrix. BES (15 mg/0.5 g of feed) was added to all the groups except the control group. Three-dimensional NMDS plot including the control group **(A)**, two-dimensional NMDS plot comparing the BES-supplemented groups **(B)**, *Q*-values resulting from pairwise multiple tests adjusted using the Benjamini–Hochberg method **(C)**. NMDS, non-metric multidimensional scaling; BES, 2-bromoethanesulfonate group; RA, reinforced acrylate pathway group; RS, reinforced succinate pathway group; CB, propionate-producing bacterial consortium group; VFA, volatile fatty acid; A:P ratio, acetate-to-propionate ratio.

Analysis of alpha and beta diversity was conducted using 16S rRNA gene long-read sequencing data from 25 rumen fluid samples, achieving 100% Good’s coverage across all samples. Alpha diversity indices, including Chao1, Shannon, and Simpson, showed no significant differences between the groups (Table 4). Beta diversity analysis based on Bray–Curtis dissimilarity demonstrated a significant distinction (*Q* < 0.001) between the CON and BES-supplemented groups. Nonetheless, the addition of microbial supplements (RA, RS, and CB) did not significantly alter the beta diversity when compared with that of the BES-only group (Figure 2), indicating that the propionate-producing bacterial consortium did not significantly shift the overall beta diversity of the rumen microbiome under conditions aimed at mitigating methane production.

**Table 4:** Alpha diversity analysis after 48 h in the *in vitro* fermentation experiment.

Alpha diversity index	Group					SEM	*p*-value
	CON	BES	BES (+)				
			RA	RS	CB		
Chao1	454	464	471	443	432	42.6	0.6015
Evenness	0.931	0.929	0.931	0.931	0.929	0.0030	0.6442
Faith’s phylogenetic diversity	27.769	26.921	27.620	26.146	25.422	1.41	0.0751
Shannon	8.212	8.218	8.267	8.180	8.129	0.12	0.4739
Simpson	0.995	0.995	0.995	0.995	0.995	0.0003	0.2661

BES was added to the RA, RS, and CB groups at the same concentration as that (15 mg/0.5 g) in the BES group.

The RA group comprised *Lactiplantibacillus plantarum* and *Megasphaera elsdenii*; RS group, *Selenomonas ruminantium* and *Acidipropionibacterium thoenii*; and CB group, all four bacterial strains.

CON, control group; BES, 2-bromoethanesulfonate group; RA, reinforced acrylate pathway group; RS, reinforced succinate pathway group; CB, propionate-producing bacterial consortium group; SM, pooled standard error of the mean.

### Investigation of multivariate association in ruminal bacteriome

**Figure 3:**
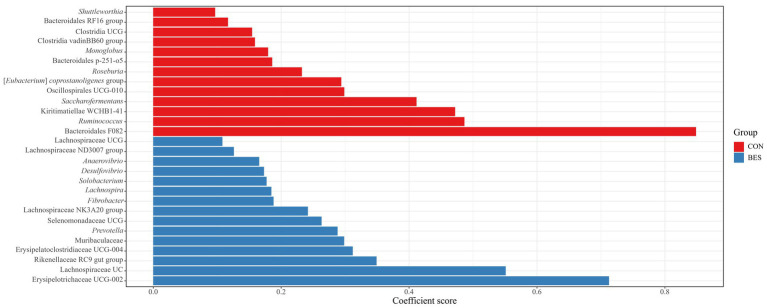
Analysis of multivariate associations in the ruminal bacteriome at the genus level between the CON and BES groups. Coefficient scores of ruminal bacteriome data showing significant differences (*Q* ≤ 0.05) between the CON and BES groups at genus level. BES, 2-bromoethanesulfonate group; UC, unclassified; UCG, uncultured genus.

**Figure 4:**
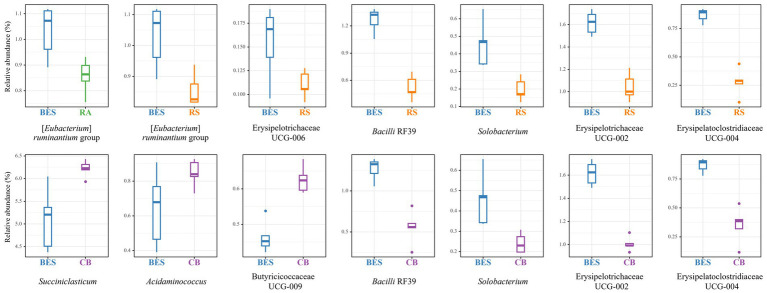
Relative abundance of major taxa at the genus level in the BES-supplementation groups. Relative abundance results of microbial taxa indicating significant differences (*Q* ≤ 0.05) between the BES, RA, RS, and CB groups at genus level. Relative abundance of major genus-level taxa with 100% occurrence in all groups among the BES-supplementation groups. RA, reinforced acrylate pathway group; RS, reinforced succinate pathway group; CB, propionate-producing bacterial consortium group; UCG, uncultured genus.

MaAsLin2 analysis at the phylum level revealed significant changes (*Q* ≤ 0.05) in the relative abundances of the major taxa present in all groups (see Supplementary Figure 1). The abundance of Verrucomicrobiota significantly dropped in the BES group (coefficient = −0.43), whereas that of Fibrobacterota and Desulfobacterota both increased (coefficients = 0.26 and 0.24, respectively). No significant differences were found among the groups receiving BES supplementation. At the genus level, 28 genera exhibited significant differences between the CON and BES groups (Figure 3). Comparing the BES group with the groups receiving additional probiotics (Figure 4) showed that one genus, [*Eubacterium*] *ruminantium* group, in the RA group; six genera in the RS group, including [*Eubacterium*] *ruminantium* group, Erysipelotrichaceae UCG-006, Bacilli RF39, *Solobacterium*, Erysipelotrichaceae UCG-002, and Erysipelatoclostridiacea UCG-004; and six genera in the CB group, including *Succiniclasticum*, *Acidaminococcus*, Butyricicoccaceae UCG-009, Bacilli RF39, *Solobacterium*, and Erysipelotrichaceae UCG-002, showed significant changes. The abundances of Bacilli RF39, *Solobacterium*, Erysipelotrichaceae UCG-002, and Erysipelatoclostridiacea UCG-004 were significantly lower in both the CB and RS groups than the abundances of those in the BES group. Additionally, the abundance of [*Eubacterium*] *ruminantium* was significantly lower in the RA and RS groups than the abundance of that in the BES group.

### Quantitative analysis using qPCR

The effects of the experimental treatments on the abundance of specific bacterial strains, total bacteria, protozoa, and methanogens in the rumen microbiome were evaluated using qPCR, and the results are summarized in Table 5. The inclusion of BES did not significantly alter the absolute abundance of total bacteria and protozoa. Similarly, the supplementation of propionate-producing bacterial strains did not significantly affect the abundance of these microbial groups. However, the inclusion of BES significantly decreased the absolute abundance of total methanogens. Excluding *M. elsdenii,* the addition of other specific bacterial strains (RA: 5.0 × 10^9^ cells/mL *L. plantarum* and *M. elsdenii*, RS: 5.0 × 10^9^ cells/mL *S. ruminantium* and *A. thoenii*, CB: 2.5 × 10^9^ cells/mL *L. plantarum*, *M. elsdenii*, *S. ruminantium*, and *A. thoenii*) caused a significant increase in the abundance of these added strains when compared with the CON and BES-only groups. The significant rise in the abundance of *A. thoenii* was directly proportional to the quantity added, underscoring the successful colonization and growth of this specific strain under the experimental conditions.

**Table 5:** Quantitative real-time polymerase chain reaction analysis for absolute quantification of microbial groups and supplemented bacterial strains.

Item	Group					SEM	*p*-value
	CON	BES	BES (+)				
			RA	RS	CB		
Absolute abundance, log copies/mL							
Total bacteria	10.30	10.26	10.33	10.27	10.34	0.06	0.1141
Total protozoa	8.97	8.96	8.97	8.94	8.97	0.01	0.9574
Total methanogen	8.17^A^	7.17^B^	7.19^B^	7.06^B^	6.89^B^	0.18	<0.0001
*Lactiplantibacillus plantarum*	1.40^C^	1.74^BC^	5.97^A^	2.41^B^	5.72^A^	0.45	<0.0001
*Megasphaera elsdenii*	7.83	7.85	8.03	7.84	7.89	0.17	0.3892
*Selenomonas ruminantium*	8.73^C^	8.76^BC^	8.91^AB^	8.93^A^	8.94^A^	0.01	0.0008
*Acidipropionibacterium thoenii*	5.00^CD^	4.87^D^	5.05^C^	6.68^A^	6.39^B^	0.07	<0.0001

CON, control group; BES, 2-bromoethanesulfonate group; RA, reinforced acrylate pathway group; RS, reinforced succinate pathway group; CB, propionate-producing bacterial consortium group; SEM, pooled standard error of the mean.

^A–D^: significant differences (*p* ≤ 0.05) between treatment groups.

## Discussion

**Figure 5:**
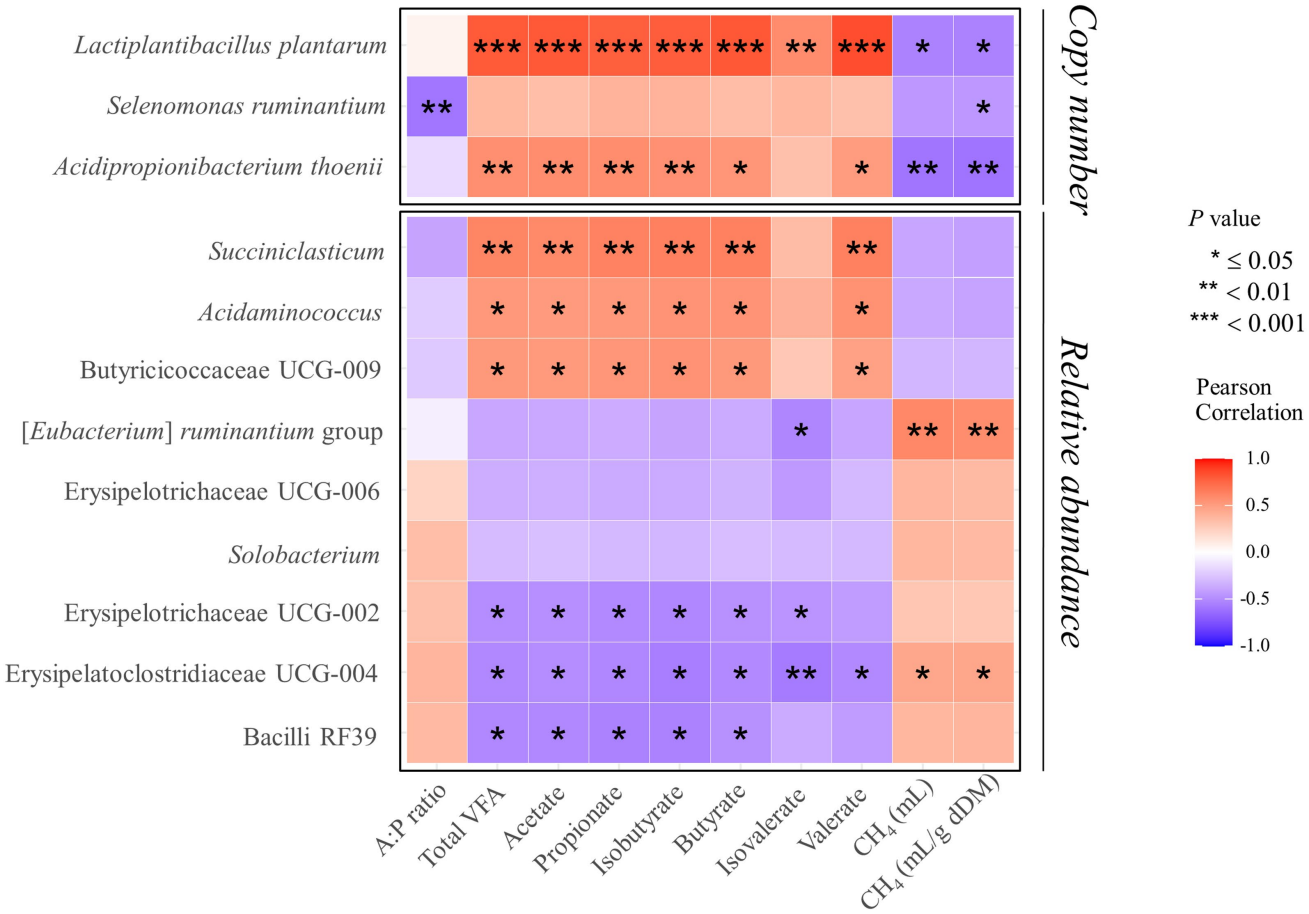
Heatmap of Pearson correlation coefficients between VFA characteristics, methane production (mL), methane yield (mL/g dDM), and three supplemented bacterial strains showing significant changes, as well as relevant bacterial genera. Significant differences are observed between the BES group and the groups supplemented with propionate-producing bacterial strains. The three supplemented bacterial strains were analyzed for correlation based on copy number, while the other relevant bacterial genera were analyzed based on relative abundance. UCG, uncultured genus; VFA, volatile fatty acid; A:P ratio, acetate-to-propionate ratio; CH_4_ (mL/g dDM), quantity of methane production per degraded gram of dry matter; *p ≤ 0.05, **p < 0.01, ***p < 0.001.

Consistent with previous studies, our findings confirmed that the addition of BES significantly reduces methanogenesis (35, 37). Moreover, the introduction of propionate-producing bacteria into a methane-mitigated environment demonstrated not only the potential for further reducing methane emissions but even the possibility of restoring total VFA in some groups (RA and CB). The acetate concentration, which contributes to H_2_ production in metabolic pathways (1), increased in the RA and CB groups beyond the levels observed in the BES-only group. The increase in acetate concentration in the RA and CB groups compared with that in the BES group indicates that microbial fermentation patterns are enhanced in a methanogen-suppressed environment. While acetate concentration was found to not have returned to the baseline levels observed in the CON group, in light of previous research finding (31) suggesting that ruminal bacteria can sense H_2_ concentrations and adjust metabolic pathways in the rumen where H_2_ has accumulated, the increase in acetate concentration observed in the RA and CB groups compared to the BES group suggests that the addition of propionate-producing bacteria may help partially resolve the issue of H_2_ accumulation. Furthermore, compared with acetate concentration, the increased butyrate concentration observed in the RA and CB groups may reflect the conversion of acetate to butyrate. This conversion also contributes to the H_2_ sink (1). The rise in propionate concentrations underscores the effective role of added bacterial strains in enhancing propionate metabolism, either directly or indirectly. The propionate production pathway does not efficiently compete with methanogenesis for metabolic H_2_ (24); hence, previous attempts to utilize propionate-producing bacteria as probiotics in standard rumen conditions *in vitro* have not yielded significant success (30). This observation suggests that methane-mitigating strategies aimed at altering the metabolic pathways of ruminal fermentation could be more effective when used in conjunction with direct inhibitors of methanogens (24). The elevation of branched-chain volatile fatty acids (BCVFAs, including isobutyrate and isovalerate) and valerate to levels comparable with those in the control group likely serves as a stimulant for cellulolytic bacteria (60), although this was not observed in our study. The addition of bacterial strains along with BES did not cause significant differences in the alpha diversity indices; however, significant shifts in beta diversity were observed. This may be attributed to the methanogenesis-inhibiting environment created by BES. The distinction in beta diversity without an alpha diversity alteration suggests that while the composition of the bacterial community changes, the overall richness and evenness of bacteriota remain stable, which indicates a balanced ecosystem adjusting to methane metabolism shifts. Ruminal bacteria are expected to fill their respective niches, thus forming a balanced ecosystem. Nevertheless, these alterations resulted in deficient VFA production in the BES-only group, which was effectively resolved by the addition of propionate-producing bacteria, as demonstrated by the effects observed in the RA and CB groups. To ascertain these pattern changes, qPCR analysis of the added bacterial strains and major microbial groups were conducted to accurately monitor select taxa in conjunction with MaAsLin2 analysis. Alongside the absence of significant differences in DMD and NDFD, no significant changes were observed in the absolute abundance of total bacteria and total protozoa. Excluding *M. elsdenii*, the addition of the other three bacterial strains significantly enhanced their abundance, suggesting their functional relevance in the BES-supplemented environment. The three bacterial strains were found to have a negative correlation with methane yield (mL/g dDM) (Figure 5). Additionally, two of the bacterial strains, *L. plantarum* and *A. thoenii*, exhibited a negative correlation with methane production (mL). *L. plantarum* and *A. thoenii* were also positively correlated with propionate concentration, which may suggest their role in enhancing propionate levels. Conversely, *S. ruminantium* displayed a negative correlation with the A:P ratio, which may support its involvement in propionate metabolism. While the bacterial strains were assisted by the direct inhibitor BES, these findings are consistent with results from previous studies (27, 30, 39), reinforcing the understanding of the effects of supplementation with these bacterial strains on methane and propionate dynamics. Moreover, BES significantly reduced the absolute abundance of methanogens, which is known to induce an imbalance in H_2_ metabolism and the accumulation of large amounts of H_2_ in the rumen (24). This prompts the growth of H_2_-utilizing bacteria, such as *Anaerovibrio* (61), *Desulfovibrio* (62), and *Fibrobacter* (27), whereas the growth of H_2_-producing cellulolytic bacteria, such as *Ruminococcus* (63) and *Saccharofermentans* (64), is lowered. The addition of BES has been shown to negatively impact the relative abundance of *Roseburia*, a bacterial genus associated with an alternative propionate-producing pathway (1). This underscores the critical role of direct inhibitors and highlights the importance of selecting suitable microbial additives to bypass their effects. The high-concentration BES treatment caused a dramatic shift in the microbial distribution between the CON and BES groups. However, there are limitations, including that the experiment was conducted only once, and the BES effect was so strong that it overshadowed the impact of both the co-treated bacterial strains and pathway-specific approaches. Despite these differences, the abundance of the [*Eubacterium*] *ruminantium* group, one of the representative ruminal fibrolytic bacteria (65), was reduced in both the RA and RS groups, suggesting that even with the use of varying probiotic strains and targeting divergent metabolic pathways, a consortium-based approach might have similar modulatory effects on the rumen microbiota. The reduction in the relative abundance of certain bacterial taxa in the RS and CB groups, including *Solobacterium*, which is known to be associated with high residual methane emissions (66), suggest that adding *S. ruminantium* and *A. thoenii*, or a propionate-producing bacterial consortium in conjunction with the methanogen-specific inhibitor, could further enhance methane reduction. When all the four probiotic strains were added in the CB group, the relative abundances of *Succiniclasticum*, *Acidaminococcus*, and Butyricicoccaceae UCG-009 significantly increased; this correlated with restoration of the total VFA profile (Figure 5). Alongside the results showing that some strains of *Succiniclasticum* and *Acidaminococcus* have a negative correlation with methane yield in dairy cows (67), the qPCR-validated successful colonization and expected fermentation outputs of supplemented bacterial strains coupled with the increased relative abundances of a major bacterial taxon *Succiniclasticum* (68), which is closely linked to propionate metabolism, demonstrate the efficacy of employing probiotic application of propionate-producing bacterial consortium for not only directing propionate production and achieving additional methane mitigation but also significantly restoring the VFA production profile in the methane-mitigated rumen environment.

In conclusion, our research findings indicate that incorporating propionate-producing bacteria is an effective biochemical approach both for augmenting methane reduction efforts and replenishing the total VFA production, which is often diminished because of abnormal H_2_ metabolism caused by the action of direct methanogenesis inhibitors *in vitro*. Bromo-compounds, such as BES, are classified as class I ozone-depleting substances (69); hence, they cannot be used for methane reduction. Instead, by integrating this strategy with the addition of available methanogen-specific inhibitors, such as 3-nitrooxypropanol (14, 70), the careful selection of probiotic candidates can further improve the availability of energy resources for the host animals. This is achieved by dual modulation of the rumen microbiome and fermentation profiles, which offers a comprehensive solution to improve both environmental and nutritional outcomes of ruminal fermentation.

## Data Availability

The datasets presented in this study can be found in online repositories. The names of the repository/repositories and accession number(s) can be found at: https://www.ncbi.nlm.nih.gov/, PRJNA1098854.

## Footnotes

^1^ https://qiime2.org/

^2^ https://www.arb-silva.de/search/testprime/

## Supplementary material

Supplementary Figure S1 Relative abundance of major bacterial taxa at the phylum level showing significant differences between the groups Relative abundance results of major phylum-level microbial taxa with 100% occurrence in all groups, indicating significant differences (Q ≤ 0.05) between the groups. CON, control group; BES, 2-bromoethanesulfonate group; RA, reinforced acrylate pathway group; RS, reinforced succinate pathway group; CB, propionate-producing bacterial consortium group; SEM, pooled standard error of the mean.

## References

[1] Wang K Xiong B Zhao X. Could propionate formation be used to reduce enteric methane emission in ruminants? Sci Total Environ. (2022) 855:158867. doi: 10.1016/j.scitotenv.2022.158867, PMID: 36122712

[2] IPCC. The Fourth Assessment Report of the Intergovernmental Panel on Climate Change. Geneva, Switzerland. (2007).

[3] Morgavi D Jouany J-P Martin C. Changes in methane emission and rumen fermentation parameters induced by Refaunation in sheep. Aust J Exp Agric. (2008) 48:69–72. doi: 10.1071/EA07236

[4] Sakthivel PC Kamra DN Agarwal N Chaudhary LC. Effect of sodium nitrate and nitrate reducing Bacteria on in vitro methane production and fermentation with Buffalo rumen liquor. Asian Australas J Anim Sci. (2012) 25:812–7. doi: 10.5713/ajas.2011.11383, PMID: 25049631 PMC4093097

[5] Doyle N Mbandlwa P Kelly WJ Attwood G Li Y Ross RP. Use of lactic acid Bacteria to reduce methane production in ruminants, a critical review. Front Microbiol. (2019) 10:2207. doi: 10.3389/fmicb.2019.02207 31632365 PMC6781651

[6] Bharanidharan R Arokiyaraj S Baik M Ibidhi R Lee SJ Lee Y. In vitro screening of east Asian plant extracts for potential use in reducing ruminal methane production. Animals. (2021) 11:1020. doi: 10.3390/ani11041020, PMID: 33916571 PMC8066825

[7] Elghandour MM Acosta-Lozano N Alvarado TD Castillo-Lopez E Cipriano-Salazar M Barros-Rodríguez M. Influence of Azadirachta Indica and *Cnidoscolus Angustidens* aqueous extract on cattle ruminal gas production and degradability in vitro. Front Vet Sci. (2023) 10:1090729. doi: 10.3389/fvets.2023.1090729, PMID: 37266386 PMC10230098

[8] Suong NTM Paengkoum S Purba RAP Paengkoum P. Optimizing anthocyanin-rich black cane (Saccharum Sinensis Robx.) silage for ruminants using molasses and Iron Sulphate: a sustainable alternative. Fermentation. (2022) 8:248. doi: 10.3390/fermentation8060248

[9] Callaway TR Carneiro De Melo AM Russell JB. The effect of Nisin and Monensin on ruminal fermentations in vitro. Curr Microbiol. (1997) 35:90–6. doi: 10.1007/s002849900218, PMID: 9216882

[10] Van Zijderveld S Gerrits W Apajalahti J Newbold J Dijkstra J Leng R. Nitrate and sulfate: effective alternative hydrogen sinks for mitigation of ruminal methane production in sheep. J Dairy Sci. (2010) 93:5856–66. doi: 10.3168/jds.2010-3281, PMID: 21094759

[11] Abrar A Kondo M Kitamura T Ban-Tokuda T Matsui H. Effect of supplementation of rice bran and fumarate alone or in combination on in vitro rumen fermentation, Methanogenesis and methanogens. Anim Sci J. (2016) 87:398–404. doi: 10.1111/asj.12431, PMID: 26388080

[12] Asanuma N Iwamoto M Hino T. Effect of the addition of fumarate on methane production by ruminal microorganisms in vitro. J Dairy Sci. (1999) 82:780–7. doi: 10.3168/jds.S0022-0302(99)75296-3, PMID: 10212465

[13] Melgar A Harper MT Oh J Giallongo F Young ME Ott TL. Effects of 3-Nitrooxypropanol on rumen fermentation, Lactational performance, and resumption of ovarian cyclicity in dairy cows. J Dairy Sci. (2020) 103:410–32. doi: 10.3168/jds.2019-17085 31733848

[14] Duin EC Wagner T Shima S Prakash D Cronin B Yáñez-Ruiz DR. Mode of action uncovered for the specific reduction of methane emissions from ruminants by the small molecule 3-nitrooxypropanol. Proc Natl Acad Sci. (2016) 113:6172–7. doi: 10.1073/pnas.1600298113, PMID: 27140643 PMC4896709

[15] Choi Y Lee SJ Kim HS Eom JS Jo SU Guan LL. Effects of seaweed extracts on in vitro rumen fermentation characteristics, methane production, and microbial abundance. Sci Rep. (2021) 11:24092. doi: 10.1038/s41598-021-03356-y, PMID: 34916562 PMC8677731

[16] Roque BM Salwen JK Kinley R Kebreab E. Inclusion of *Asparagopsis Armata* in lactating dairy cows’ diet reduces enteric methane emission by over 50 percent. J Clean Prod. (2019) 234:132–8. doi: 10.1016/j.jclepro.2019.06.193

[17] Appuhamy JRN Strathe A Jayasundara S Wagner-Riddle C Dijkstra J France J. Anti-methanogenic effects of Monensin in dairy and beef cattle: a meta-analysis. J Dairy Sci. (2013) 96:5161–73. doi: 10.3168/jds.2012-5923, PMID: 23769353

[18] Lovett D Lovell S Stack L Callan J Finlay M Conolly J. Effect of forage/concentrate ratio and dietary coconut oil level on methane output and performance of finishing beef heifers. Livest Prod Sci. (2003) 84:135–46. doi: 10.1016/j.livprodsci.2003.09.010

[19] Wu H Meng Q Yu Z. Effect of Ph buffering capacity and sources of dietary sulfur on rumen fermentation, sulfide production, methane production, sulfate reducing bacteria, and total archaea in in vitro rumen cultures. Bioresour Technol. (2015) 186:25–33. doi: 10.1016/j.biortech.2015.02.110, PMID: 25797103

[20] Cottle DJ Nolan JV Wiedemann SG. Ruminant enteric methane mitigation: a review. Anim Prod Sci. (2011) 51:491–514. doi: 10.1071/AN10163

[21] Kumar S Puniya AK Puniya M Dagar SS Sirohi SK Singh K. Factors affecting rumen methanogens and methane mitigation strategies. World J Microbiol Biotechnol. (2009) 25:1557–66. doi: 10.1007/s11274-009-0041-3

[22] Henderson G Cox F Ganesh S Jonker A Young W Janssen PH. Rumen microbial community composition varies with diet and host, but a core microbiome is found across a wide geographical range. Sci Rep. (2015) 5:14567. doi: 10.1038/srep14567, PMID: 26449758 PMC4598811

[23] Li J Zhao S Meng Z Gao Y Miao J Mao S. Effects of fumarate and nitroglycerin on in vitro rumen fermentation, methane and hydrogen production, and on microbiota. Biology. (2023) 12:1011. doi: 10.3390/biology12071011, PMID: 37508440 PMC10376899

[24] Ungerfeld EM. Metabolic hydrogen flows in rumen fermentation: principles and possibilities of interventions. Front Microbiol. (2020) 11:589. doi: 10.3389/fmicb.2020.00589, PMID: 32351469 PMC7174568

[25] Zhao Y Zhao G. Decreasing ruminal methane production through enhancing the sulfate reduction pathway. Anim Nutr. (2022) 9:320–6. doi: 10.1016/j.aninu.2022.01.006 35600554 PMC9097629

[26] Ellis J Dijkstra J Kebreab E Bannink A Odongo N McBride B. Aspects of rumen microbiology central to mechanistic modelling of methane production in cattle. J Agric Sci. (2008) 146:213–33. doi: 10.1017/S0021859608007752

[27] Jeyanathan J Martin C Morgavi DP. The use of direct-fed microbials for mitigation of ruminant methane emissions: a review. Animal. (2014) 8:250–61. doi: 10.1017/S1751731113002085, PMID: 24274095

[28] Danielsson R Dicksved J Sun L Gonda H Müller B Schnürer A. Methane production in dairy cows correlates with rumen methanogenic and bacterial community structure. Front Microbiol. (2017) 8:226. doi: 10.3389/fmicb.2017.00226, PMID: 28261182 PMC5313486

[29] Alazzeh A Sultana H Beauchemin K Wang Y Holo H Harstad O. Using strains of propionibacteria to mitigate methane emissions in vitro. Acta Agric Scand A Anim Sci. (2012) 62:263–72. doi: 10.1080/09064702.2013.773056

[30] Chen J Harstad OM McAllister T Dörsch P Holo H. Propionic acid bacteria enhance ruminal feed degradation and reduce methane production in vitro. Acta Agric Scandinavica Sect A Anim Sci. (2020) 69:169–75. doi: 10.1080/09064702.2020.1737215

[31] Greening C Geier R Wang C Woods LC Morales SE McDonald MJ. Diverse hydrogen production and consumption pathways influence methane production in ruminants. ISME J. (2019) 13:2617–32. doi: 10.1038/s41396-019-0464-2, PMID: 31243332 PMC6776011

[32] Smith PE Kelly AK Kenny DA Waters SM. Differences in the composition of the rumen microbiota of finishing beef cattle divergently ranked for residual methane emissions. Front Microbiol. (2022) 13:855565. doi: 10.3389/fmicb.2022.855565, PMID: 35572638 PMC9099143

[33] Kamke J Kittelmann S Soni P Li Y Tavendale M Ganesh S. Rumen metagenome and Metatranscriptome analyses of low methane yield sheep reveals a Sharpea-enriched microbiome characterised by lactic acid formation and utilisation. Microbiome. (2016) 4:1–16. doi: 10.1186/s40168-016-0201-2, PMID: 27760570 PMC5069950

[34] El Hage R Hernandez-Sanabria E Calatayud Arroyo M Props R Van de Wiele T. Propionate-producing consortium restores antibiotic-induced Dysbiosis in a dynamic in vitro model of the human intestinal microbial ecosystem. Front Microbiol. (2019) 10:1206. doi: 10.3389/fmicb.2019.01206, PMID: 31214145 PMC6554338

[35] Pellikaan W Hendriks W Uwimana G Bongers L Becker P Cone J. A novel method to determine simultaneously methane production during in vitro gas production using fully automated equipment. Anim Feed Sci Technol. (2011) 168:196–205. doi: 10.1016/j.anifeedsci.2011.04.096

[36] Fonty G Joblin K Chavarot M Roux R Naylor G Michallon F. Establishment and development of ruminal Hydrogenotrophs in methanogen-free lambs. Appl Environ Microbiol. (2007) 73:6391–403. doi: 10.1128/AEM.00181-07, PMID: 17675444 PMC2075041

[37] Ungerfeld E Rust S Boone D Liu Y. Effects of several inhibitors on pure cultures of ruminal methanogens. J Appl Microbiol. (2004) 97:520–6. doi: 10.1111/j.1365-2672.2004.02330.x 15281932

[38] Stewart C. Lactic acid bacteria in the rumen. Lactic Acid Bacteria. (1992) 1:49–68. doi: 10.1007/978-1-4615-3522-5_3

[39] Zhang X Franco M Kharazian ZA Khan A Zhang J Ding Z. *Lactiplantibacillus plantarum* Bx62 reduces methane production, and improves antioxidant capacity and rumen fermentation in vitro. Anim Feed Sci Technol. (2023) 300:115655. doi: 10.1016/j.anifeedsci.2023.115655

[40] Hino T Kuroda S. Presence of lactate dehydrogenase and lactate racemase in *Megasphaera Elsdenii* grown on glucose or lactate. Appl Environ Microbiol. (1993) 59:255–9. doi: 10.1128/aem.59.1.255-259.1993, PMID: 8439152 PMC202087

[41] Reichardt N Duncan SH Young P Belenguer A McWilliam Leitch C Scott KP. Phylogenetic distribution of three pathways for propionate production within the human gut microbiota. ISME J. (2014) 8:1323–35. doi: 10.1038/ismej.2014.14, PMID: 24553467 PMC4030238

[42] Mira P Yeh P Hall BG. Estimating microbial population data from optical density. PLoS One. (2022) 17:e0276040. doi: 10.1371/journal.pone.0276040, PMID: 36228033 PMC9562214

[43] Parte A Whitman WB Goodfellow M Kämpfer P Busse H-J Trujillo ME. Bergey’s manual of systematic bacteriology: the actinobacteria, vol. 5. New York: Springer (2012).

[44] Vos P Garrity G Jones D Krieg NR Ludwig W Rainey FA. Bergey’s manual of systematic bacteriology: the firmicutes. New York: Springer (2011).

[45] Haya S Tokumaru Y Abe N Kaneko J Aizawa S-i. Characterization of lateral flagella of *Selenomonas Ruminantium*. Appl Environ Microbiol. (2011) 77:2799–802. doi: 10.1128/AEM.00286-11, PMID: 21335384 PMC3126368

[46] Loferer-Krossbacher M Klima J Psenner R. Determination of bacterial cell dry mass by transmission electron microscopy and densitometric image analysis. Appl Environ Microbiol. (1998) 64:688–94. doi: 10.1128/AEM.64.2.688-694.1998, PMID: 9464409 PMC106103

[47] Goering HK. Forage fiber analyses (apparatus, reagents, procedures, and some applications) USDA, Washington, D.C., USA: Agricultural Handbook (1970).

[48] Theodorou MK Williams BA Dhanoa MS McAllan AB France J. A simple gas production method using a pressure transducer to determine the fermentation kinetics of ruminant feeds. Anim Feed Sci Technol. (1994) 48:185–97. doi: 10.1016/0377-8401(94)90171-6

[49] Hamid MMA Moon J Yoo D Kim H Lee YK Song J. Rumen fermentation, methane production, and microbial composition following in vitro evaluation of red ginseng byproduct as a protein source. J Anim Sci Technol. (2020) 62:801–11. doi: 10.5187/jast.2020.62.6.801, PMID: 33987561 PMC7721587

[50] Yáñez-Ruiz DR Bannink A Dijkstra J Kebreab E Morgavi DP O’Kiely P. Design, implementation and interpretation of in vitro batch culture experiments to assess enteric methane mitigation in ruminants—a review. Anim Feed Sci Technol. (2016) 216:1–18. doi: 10.1016/j.anifeedsci.2016.03.016

[51] Chaney AL Marbach EP. Modified reagents for determination of urea and Ammonia. Clin Chem. (1962) 8:130–2. doi: 10.1093/clinchem/8.2.130, PMID: 13878063

[52] Yu Z Morrison M. Improved extraction of Pcr-quality community DNA from Digesta and fecal samples. BioTechniques. (2004) 36:808–12. doi: 10.2144/04365ST04, PMID: 15152600

[53] Bolyen E Rideout JR Dillon MR Bokulich NA Abnet CC Al-Ghalith GA. Reproducible, interactive, scalable and extensible microbiome data science using Qiime 2. Nat Biotechnol. (2019) 37:852–7. doi: 10.1038/s41587-019-0209-9, PMID: 31341288 PMC7015180

[54] Callahan BJ McMurdie PJ Rosen MJ Han AW Johnson AJA Holmes SP. Dada2: high-resolution sample inference from Illumina amplicon data. Nat Methods. (2016) 13:581–3. doi: 10.1038/nmeth.3869, PMID: 27214047 PMC4927377

[55] Quast C Pruesse E Yilmaz P Gerken J Schweer T Yarza P. The Silva ribosomal Rna gene database project: improved data processing and web-based tools. Nucleic Acids Res. (2012) 41:D590–6. doi: 10.1093/nar/gks1219 23193283 PMC3531112

[56] Cameron ES Schmidt PJ Tremblay BJM Emelko MB Müller KM. Enhancing diversity analysis by repeatedly rarefying next generation sequencing data describing microbial communities. Sci Rep. (2021) 11:22302. doi: 10.1038/s41598-021-01636-1, PMID: 34785722 PMC8595385

[57] Klindworth A Pruesse E Schweer T Peplies J Quast C Horn M. Evaluation of general 16s ribosomal Rna gene Pcr primers for classical and next-generation sequencing-based diversity studies. Nucleic Acids Res. (2013) 41:e1. doi: 10.1093/nar/gks808, PMID: 22933715 PMC3592464

[58] Oksanen J. Vegan: community ecology package. (2010). Available at: http://vegan.r-forge.r-project.org/.

[59] Mallick H Rahnavard A McIver LJ Ma S Zhang Y Nguyen LH. Multivariable association discovery in population-scale meta-omics studies. PLoS Comput Biol. (2021) 17:e1009442. doi: 10.1371/journal.pcbi.1009442, PMID: 34784344 PMC8714082

[60] Roman-Garcia Y Mitchell KE Lee C Socha MT Park T Wenner BA. Conditions stimulating neutral detergent fiber degradation by dosing branched-chain volatile fatty acids. Iii: relation with solid passage rate and Ph on prokaryotic fatty acid profile and community in continuous culture. J Dairy Sci. (2021) 104:9868–85. doi: 10.3168/jds.2021-20336, PMID: 34253360

[61] Henderson C. The influence of extracellular hydrogen on the metabolism of *Bacteroides ruminicola*, *Anaerovibrio lipolytica*, and *Selenomonas ruminantium*. J Gen Microbiol. (1980) 119:485–91. doi: 10.1099/00221287-119-2-485 6785381

[62] Zhao Y Xie B Gao J Zhao G. Dietary supplementation with sodium sulfate improves rumen fermentation, fiber digestibility, and the plasma metabolome through modulation of rumen bacterial communities in steers. Appl Environ Microbiol. (2020) 86:e01412-20. doi: 10.1128/AEM.01412-20 32859601 PMC7642074

[63] Miller TL Wolin M. Formation of hydrogen and formate by *Ruminococcus albus*. J Bacteriol. (1973) 116:836–46. doi: 10.1128/jb.116.2.836-846.1973, PMID: 4745433 PMC285454

[64] Jin Y Huang Y Luo H Wang L Chen B Zhang Y. Effects of replacing hybrid Giant Napier with sugarcane bagasse and fermented sugarcane bagasse on growth performance, nutrient digestibility, rumen fermentation characteristics, and rumen microorganisms of Simmental crossbred cattle. Front Microbiol. (2023) 14:1236955. doi: 10.3389/fmicb.2023.1236955, PMID: 38045032 PMC10693430

[65] Koike S Kobayashi Y. Fibrolytic rumen bacteria: their ecology and functions. Asian Australas J Anim Sci. (2009) 22:131–8. doi: 10.5713/ajas.2009.r.01

[66] Andrade BG Bressani FA Cuadrat RR Cardoso TF Malheiros JM de Oliveira PS. Stool and ruminal microbiome components associated with methane emission and feed efficiency in Nelore beef cattle. Front Genet. (2022) 13:812828. doi: 10.3389/fgene.2022.812828, PMID: 35656319 PMC9152269

[67] Savin KW Moate PJ Williams S Bath C Hemsworth J Wang J. Dietary wheat and reduced methane yield are linked to rumen microbiome changes in dairy cows. PLoS One. (2022) 17:e0268157. doi: 10.1371/journal.pone.0268157, PMID: 35587477 PMC9119556

[68] Van Gylswyk N. *Succiniclasticum ruminis* gen. nov., sp. nov., a ruminal bacterium converting succinate to propionate as the sole energy-yielding mechanism. Int J Syst Evol Microbiol. (1995) 45:297–300. doi: 10.1099/00207713-45-2-297 7537062

[69] Hristov AN Oh J Firkins JL Dijkstra J Kebreab E Waghorn G. Special topics--mitigation of methane and nitrous oxide emissions from animal operations: I. A review of enteric methane mitigation options. J Anim Sci. (2013) 91:5045–69. doi: 10.2527/jas.2013-6583 24045497

[70] Pitta DW Indugu N Melgar A Hristov A Challa K Vecchiarelli B. The effect of 3-nitrooxypropanol, a potent methane inhibitor, on ruminal microbial gene expression profiles in dairy cows. Microbiome. (2022) 10:1–21. doi: 10.1186/s40168-022-01341-9 36100950 PMC9469553

