# Supplementary figures and images for: Application of propionate-producing bacterial consortium in ruminal methanogenesis inhibited environment with bromoethanesulfonate as a methanogen direct inhibitor
Source: Front Vet Sci. 2024 Oct 9;11:1422474. doi: 10.3389/fvets.2024.1422474 (PMC11497462; doi:10.3389/fvets.2024.1422474)

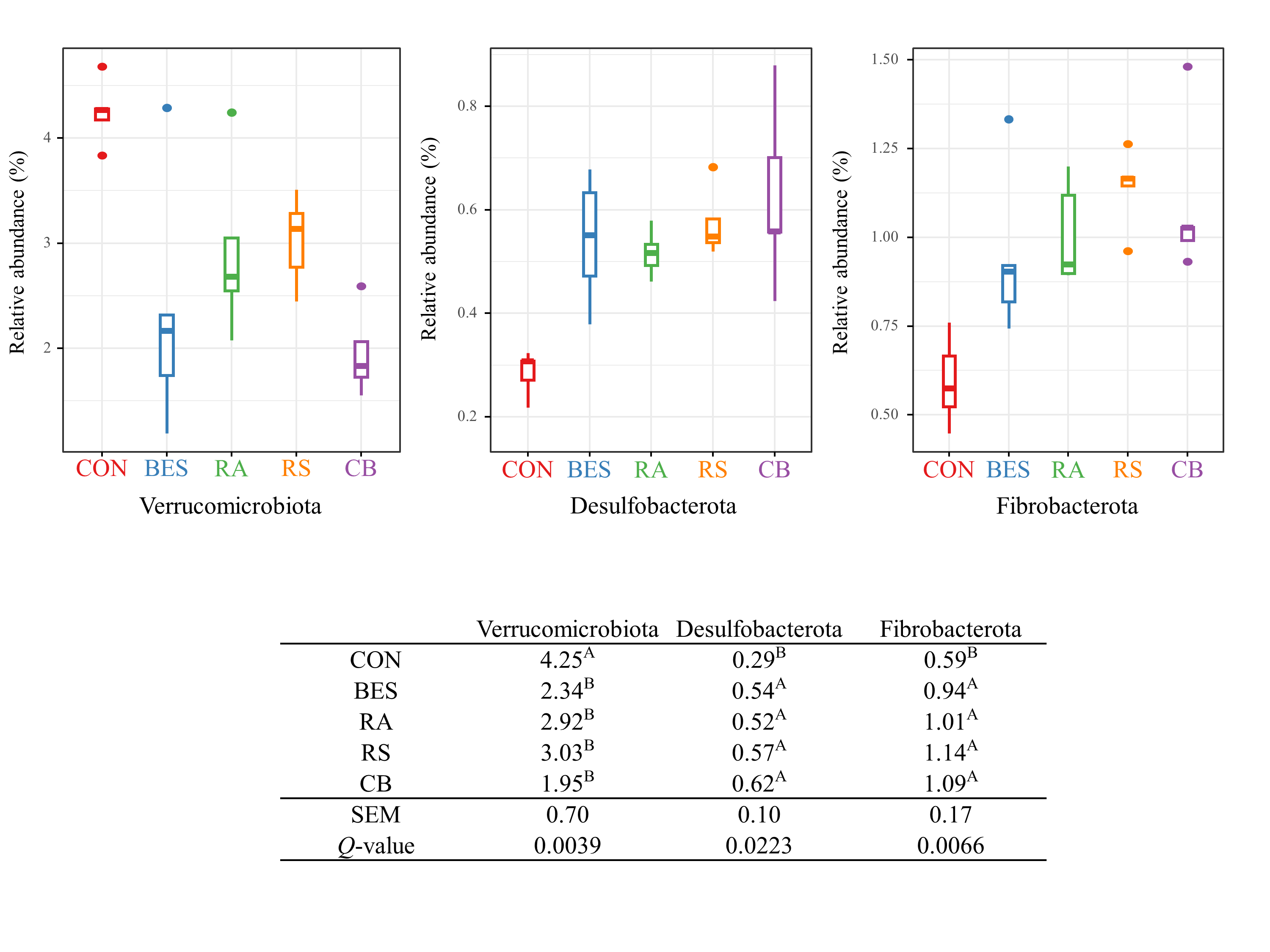

Supplement: Supplementary Figure S1 — Relative abundance of major bacterial taxa at the phylum level showing significant differences between the groups Relative abundance results of major phylum-level microbial taxa with 100% occurrence in all groups, indicating significant differences (Q ≤ 0.05) between the groups. CON, control group; BES, 2-bromoethanesulfonate group; RA, reinforced acrylate pathway group; RS, reinforced succinate pathway group; CB, propionate-producing bacterial consortium group; SEM, pooled standard error of the mean. [file Image_1.tif]
