# Supplementary material for: Application of propionate-producing bacterial consortium in ruminal methanogenesis inhibited environment with bromoethanesulfonate as a methanogen direct inhibitor
Source: Front Vet Sci. 2024 Oct 9;11:1422474. doi: 10.3389/fvets.2024.1422474 (PMC11497462; doi:10.3389/fvets.2024.1422474)
Supplement: Supplementary file 2 [file Table_1.docx]

Supplementary Table 1. Primer set used *in vitro* digestibility experiment

| Species or microbial group | Target gene | Primer name | Sequence (5'-3') | Amplicon size (bp) | PCR condition | Reference | Reference genome |
| --- | --- | --- | --- | --- | --- | --- | --- |
| *Lactiplantibacillus plantarum* | Unique gene | Plantarum-F | GCTGGCAATGCCATCGTGCT | 128 | 95°C (2 min) + 95°C (5 s) + 60°C (30 s) | (1) | *L. plantarum* |
|  |  | Plantarum-R | TCTCAACGGTTGCTGTATCG |  |  |  |  |
| *Megasphaera elsdenii* | 16S rRNA gene | MegEls2F | AGATGGGGACAACAGCTGGA | 95 | 95°C (10 min) + 95°C (15 s) + 59°C (60 s) | (2) | *M. elsdenii* |
|  |  | MegEls2R | CGAAAGCTCCGAAGAGCCT |  |  |  |  |
| *Selenomonas ruminantium* | 16S rRNA gene | SelRum1F | GGCGGGAAGGCAAGTCAGTC | 83 | 95°C (10 min) + 95°C (15 s) + 60°C (60 s) | (3) | *S. ruminantium* |
|  |  | SelRum1R | CCTCTCCTGCACTCAAGAAAGACAG |  |  |  |  |
| *Acidipropionibacterium thoenii* | Unique gene | Pth_aroE-F3 | CGCCGAGAACCTGCCCGAGT | 128 | 95°C (10 min) + 95°C (15 s) + 63°C (60 s) | (4) | *A. thoenii* |
|  |  | Pth_aroE-R3 | TGGGCCAGGGATCGTAGATG |  |  |  |  |
| Total bacteria | 16S rRNA gene | 27f | AGAGTTTGATCMTGGCTCAG | 1493 | 94°C (10 min) + 94°C (30 s) + 55°C (60 s) + 72°C (90 s) | (5) | *Prevotella ruminicola* |
|  |  | 1492r | TACGGYTACCTTGTTACGACTT |  |  |  |  |
|  | 16S rRNA gene | 340f | TCCTACGGGAGGCAGCAGT | 467 | 95°C (10 min) + 95°C (15 s) + 60°C (60 s) | (6) |  |
|  |  | 806r | GGACTACCAGGGTATCTAATCCTGTT |  |  |  |  |
| Total protozoa | 18S rRNA gene | P.SSU-54f | CAYGTCTAAGTATAAATAACTAC | 1536 | 94°C (10 min) + 94°C (45 s) + 55 (45 s) + 72 (90 s) | (7) | *Entodinium caudatum* |
|  |  | P.SSU-1747r | CTCTAGGTGATWWGRTTTAC |  |  |  |  |
|  | 18S rRNA gene | 316f | GCTTTCGWTGGTAGTGTATT | 234 | 95°C (10 min) + 94°C (30 s) + 54°C (30 s) + 72°C (60 s) | (7) |  |
|  |  | 539r | CTTGCCCTCYAATCGTWCT |  |  |  |  |
| Total methanogen | 16S rRNA gene | Met86f | GCTCAGTAACACGTGG | 791 | 95°C (10 min) + 95°C (15 s) + 55°C (30 s) + 72°C (60 s) | (8) | *Methanobrevibacter ruminantium* |
|  |  | Met915r | GTGCTCCCCCGCCAATTCCT |  |  |  |  |
| Unique gene *L. plantarum*, LPXTG-motif cell wall anchor domain protein; *A. thoenii*, shikimate 5-dehydrogenase Reference genome *L. plantarum,* EFK29584.1; *M. elsdenii,* NR_029207.1; *S. ruminantium,* NR_044734.1; *A. thoenii*, WP_027586906.1; Total bacteria, AJ009933.1; Total protozoa, AM158447.1; Total methanogen, NR_042784.1 | | | | | | | |

References

1. Kim E, Yang S-M, Lim B, Park SH, Rackerby B, Kim H-Y. Design of Pcr Assays to Specifically Detect and Identify 37 Lactobacillus Species in a Single 96 Well Plate. *BMC microbiol.* (2020) 20:1-14.

2. Stevenson DM, Weimer PJ. Dominance of Prevotella and Low Abundance of Classical Ruminal Bacterial Species in the Bovine Rumen Revealed by Relative Quantification Real-Time Pcr. *Appl. microbiol. and biotechnol.* (2007) 75:165-74.

3. Khafipour E, Li S, Plaizier JC, Krause DO. Rumen Microbiome Composition Determined Using Two Nutritional Models of Subacute Ruminal Acidosis. *Appl Environ. Microbiol.* (2009) 75(22):7115-24.

4. Turgay M, Schaeren W, Wechsler D, Bütikofer U, Graber HU. Fast Detection and Quantification of Four Dairy Propionic Acid Bacteria in Milk Samples Using Real-Time Quantitative Polymerase Chain Reaction. *Inter. Dairy J.* (2016) 61:37-43.

5. Lane D. 16s/23s Rrna Sequencing. *Nucleic acid techniques in bacterial systematics* (1991).

6. Nadkarni MA, Martin FE, Jacques NA, Hunter N. Determination of Bacterial Load by Real-Time Pcr Using a Broad-Range (Universal) Probe and Primers Set. *Microbiol* (2002) 148(1):257-66.

7. Sylvester JT, Karnati SK, Yu Z, Morrison M, Firkins JL. Development of an Assay to Quantify Rumen Ciliate Protozoal Biomass in Cows Using Real-Time Pcr. *J. nutr.* (2004) 134(12):3378-84.

8. Danielsson R, Schnürer A, Arthurson V, Bertilsson J. Methanogenic Population and Ch4 Production in Swedish Dairy Cows Fed Different Levels of Forage. *Appl Environ Microbiol* (2012) 78(17):6172-9.
